# *Bacillus anthracis* Interacts with Plasmin(ogen) to Evade C3b-Dependent Innate Immunity

**DOI:** 10.1371/journal.pone.0018119

**Published:** 2011-03-25

**Authors:** Myung-Chul Chung, Jessica H. Tonry, Aarthi Narayanan, Nathan P. Manes, Ryan S. Mackie, Bradford Gutting, Dhritiman V. Mukherjee, Taissia G. Popova, Fatah Kashanchi, Charles L. Bailey, Serguei G. Popov

**Affiliations:** 1 National Center for Biodefense and Infectious Diseases, George Mason University, Manassas, Virginia, United States of America; 2 Chemical, Biological and Radiological Defense Division, Naval Surface Warfare Center, Dahlgren Division, Dahlgren, Virginia, United States of America; Max Planck Institute for Infection Biology, Germany

## Abstract

The causative agent of anthrax, *Bacillus anthracis*, is capable of circumventing the humoral and innate immune defense of the host and modulating the blood chemistry in circulation to initiate a productive infection. It has been shown that the pathogen employs a number of strategies against immune cells using secreted pathogenic factors such as toxins. However, interference of *B. anthracis* with the innate immune system through specific interaction of the spore surface with host proteins such as the complement system has heretofore attracted little attention. In order to assess the mechanisms by which *B. anthracis* evades the defense system, we employed a proteomic analysis to identify human serum proteins interacting with *B. anthracis* spores, and found that plasminogen (PLG) is a major surface-bound protein. PLG efficiently bound to spores in a lysine- and exosporium-dependent manner. We identified α-enolase and elongation factor tu as PLG receptors. PLG-bound spores were capable of exhibiting anti-opsonic properties by cleaving C3b molecules *in vitro* and in rabbit bronchoalveolar lavage fluid, resulting in a decrease in macrophage phagocytosis. Our findings represent a step forward in understanding the mechanisms involved in the evasion of innate immunity by *B. anthracis* through recruitment of PLG resulting in the enhancement of anti-complement and anti-opsonization properties of the pathogen.

## Introduction

*Bacillus anthracis*, the causative organism of anthrax, is a spore-forming Gram-positive bacterium. Infection can be initiated by the spores through inhalation, ingestion, or cutaneous abrasions. In inhalational infection, alveolar macrophages phagocytose the spores deposited on the lung surface and deliver them to the regional lymph nodes. In spite of the bactericidal activity of macrophages, some of the engulfed spores survive, germinate into vegetative bacteria, kill the macrophage, and subsequently become released into the lymphatic system. Further growth of the bacteria leads to the hemorrhagic lymphadenitis, allowing the pathogen to break into the bloodstream and spread systemically through the circulation [1], [2].

In order to initiate a productive infection, *B. anthracis* needs to circumvent the innate protective response of the host. It has been shown that the pathogen employs a number of strategies against the immune cells using secreted pathogenic factors. For example, lethal and edema toxins secreted along with other virulence factors such as hemolysins within the phagosomal compartment of macrophages allow the bacteria to resist being killed and to escape from the phagocytes [3]. Cleavage of mitogen-activated protein kinase kinases by lethal toxin seems to play a central role in the immunosuppressive capacity of *B. anthracis* to induce necrosis or apoptosis of macrophages [4] and to inhibit responses of dendritic and T cells [5]–[7]. The pore-forming hemolysin anthrolysin O is able to damage membranes of different immune cell types and to sensitize macrophages to lethal toxin [8]. However, interference of *B. anthracis* with the innate immune system through specific interaction of the spore surface with the host proteins such as the complement system has heretofore attracted little attention.

The complement system facilitates bactericidal activity of normal human serum (NHS) in early clearance of pathogens [9], [10]. The complement system can be activated through three different pathways: classical, lectin, and alternative. Deposition of complement C3b onto the bacterial surface is a crucial step in eliminating the pathogen. To escape complement-mediated killing, pathogens use a common evasion strategy by acquiring the fluid-phase complement factor H, complement factor H-related proteins (FHRs), complement factor H-like proteins (FHLs), the complement C4-binding protein from host serum [10], [11]. It has also been observed that *B. anthracis* can directly infect non-phagocytic cells [12] and invade tissues of the nasopharynx after spore inhalation without needing to be transported by alveolar macrophages to the lymphatics [13].

A number of pathogens bind host zymogen protease plasminogen (PLG) to the bacterial surface for tissue invasion [14]. PLG is an abundant protein found in the plasma and is a central component of the fibrinolytic system. Activation of the fibrinolytic system by PLG has recently been found during *B. anthracis* infection in mice [15]. In the host, inactive PLG is converted to active plasmin by host-expressed tissue-type PLG activator (tPA) and urokinase (uPA). PLG activation to plasmin by invasive pathogenic bacteria such as *Borrelia burgdorferi* [16] or *Pseudomonas aeruginosa* [17] could substantially augment the organism's potential for tissue invasion and necrosis. However, *B. anthracis* protease InhA can accelerate the uPA-mediated plasminogen activation, thereby suggesting a mechanism of plasmin modulation in anthrax infection [15]. As a component of the exosporium [18], this protease might be relevant to the invasive properties of the spores. The active plasmin is a broad-spectrum serine protease that dominantly degrades non-collagenous extracellular matrix (ECM) and basal membrane proteins such as laminin and fibronectin [19]. A recent study also showed that plasmin bound to the borrelial surface leads to a drastic decrease in C3b deposition, suggesting that plasmin has anti-opsonic properties [16].

Since functional complement proteins are present in the bronchoalveolar lavage fluid (BALF) [20], complement C3-dependent opsonization is expected to play an important role in the early stages of inhalational *B. anthracis* infection. In fact, C3b can bind to *B. anthracis* spores opsonized by the normal human serum (NHS) and thus enhance phagocytosis by human macrophages [21]. Complement-deficient A/J mice are highly susceptible to the attenuated *B. anthracis* Sterne strain [22], and the resistant C57BL/6 mice acquire susceptibility to challenge with the attenuated Sterne strain after depletion of complement by cobra venom injection [23].

The above considerations prompted us to investigate the proteome of NHS bound to *B. anthracis* spores. Here we provide evidence that PLG binds *B. anthracis* through surface α-enolase and elongation factor-tu, and its activation to plasmin by uPA results in a reduction in C3b/iC3b deposition in spores. Recent studies suggested that bacterial surface proteins α–enolase and glyceraldehyde-3-phasphate dehydrogenase of *B. anthracis* bound PLG [24], [25]. Together with the results, our findings represent a step forward in understanding the mechanisms involved in *B. anthracis* resistance to complement attack and opsonization resulting in the increased ability of the pathogen to invade the host.

## Materials and Methods

### Bacterial strains and reagents

*B. anthracis* non-encapsulated Sterne strain 34F2 [pXO1^+^, pXO2^−^] was obtained from the Colorado Serum Company. Non-virulent *B. subtilis* 168 was purchased from the American Type Culture Collection (Manassas, VA, USA). Human PLG, plasmin, rabbit anti-GroEL polyclonal antibody, and D-Val-Leu-Lys-*p*-nitroanilide (VLK-pNA, Sigma V7127) were purchased from Sigma. Human complement C3b (product # 204860) and uPA (product # 672112) were purchased from Calbiochem, and rabbit anti-human C3c polyclonal antibody (product # A0062) was from Dako. NHS was obtained from Innovative and goat anti-human PLG polyclonal antibody (ab6189) and rabbit anti-mouse C3 polyclonal antibody (ab11887) from Abcam. Mouse monoclonal antibody (EF12) against an abundant *B. anthracis* exosporium protein BclA (1 mg/ml; used 1∶1,000 dilution for Western blot) [26] was kindly provided by J.F. Kearney (University of Alabama). Horse raddish peroxidase (HRP)-conjugated secondary antibodies sheep anti-mouse IgG and donkey anti-rabbit IgG antibody were purchased from GE Healthcare and rabbit anti-goat IgG antibody from Jackson Immuno Research.

### PLG binding onto *B. anthracis*

1.6×10^9^ spores or 100 µl of vegetative cells grown to A_600_ of 1.5 were incubated with 150 µl of NHS or 10 µg of purified PLG in binding buffer (50 mM Tris, pH 7.5, 100 mM NaCl, and 2 mM MgCl_2_) for 1 h at room temperature on a rocker platform and then washed 4 times with binding buffer. The 5^th^ wash in 100 µl of binding buffer was saved as a wash control, and bound proteins were eluted with 100 µl of 3 M potassium thiocyanate. The eluted protein was separated by SDS-PAGE and analyzed by Western blot with anti-PLG. To examine the effects of amino acids on PLG binding to the pathogen surface, 5×10^7^ Sterne spores in binding buffer were incubated with 2 µg of PLG and 50 mM amino acids for 1 h on a rocker platform. The spores were washed 2 times with 1 ml of binding buffer and incubated in 150 µl of 50 mM Tris-HCl, pH 7.5, complemented with 20 units (0.2 ug) of uPA and 150 µl of 200 µM VLK-pNA for 15 min at 37°C. Plasmin activity was measured by reading absorbance of 100 µl of reactants at 405 nm. The spores were suspended in 150 µl of binding buffer and incubated with 4 µg of PLG for 1 h. After 5 washings with 150 µl of the binding buffer, the spores were suspended in 300 µl of 50 mM Tris-HCl, pH 7.5, and incubated with 20 units of uPA and substrate. Plasmin activity was measured as described above.

### Two-Dimensional SDS-PAGE

Spore-bound NHS proteins were eluted by a chaotropic reagent, potassium thiocyanate, as described above and dialyzed in water. Samples were subjected to two-dimensional (2D) electrophoresis as follows: the desalted proteins were dissolved in Zoom 2D protein solubilizer-1 and applied on immobilized pH 3–10 linear gradient strips according to the manufacturer's instructions (Invitrogen). Focusing started at 175 V (15 min), was ramped to 2000 V for 45 min, and finally continued at 2000 V for 30 min in an IPGrunner system (Invitrogen). After focusing, strips were equilibrated for sample buffer and then overlaid onto 4–12% SDS-PAGE. The separated proteins were silver-stained and the bands were excised from the stained gel.

### Mass spectrometry

The potassium thiocyanate-eluted spore-bound proteins or silver-stained protein bands excised from the 2D gel were trypsinized as described [15]. Identification of the proteins was performed by LTQ-tandem MS/MS equipped with a reverse-phase liquid chromatography nanospray tandem MS using a high-resolution LTQ-Orbitrap spectrometer (ThermoFisher). The reverse-phase column was slurry-packed in house with 5 µm, 200-Å pore size C_18_ resin (Michrom BioResources) in a 100 µm×10 cm fused silica capillary (Polymicro Technologies) with a laser-pulled tip. After sample injection, the column was washed for 5 min at 200 nl/min with 0.1% formic acid, peptides were eluted using a 50-min linear gradient from 0 to 40% acetonitrile and an additional step of 80% acetonitrile (all in 0.1% formic acid) for 5 min. The LTQ-Orbitrap MS was operated in a data-dependent mode in which each full MS scan was followed by five MS-MS scans where the five most abundant molecular ions were dynamically selected and fragmented by collision-induced dissociation using normalized collision energy of 35%. Tandem mass spectra were matched against the National Center for Biotechnology Information mouse database by Sequest Bioworks software (ThermoFisher) using full tryptic cleavage constraints and static cysteine alkylation by iodoacetamide. For a peptide to be considered legitimately identified, it had to be the top number one matched and had to achieve cross-correlation scores of 1.9 for [M+H]^1+^, 2.2 for [M+2H]^2+^, 3.5 for [M+3H]^3+^, ΔCn>0.1, and a maximum probability of randomized identification of 0.01. The MS data were filtered to improve the quality of the data set prior to protein selection. The initial set of proteins was limited to those that could be confidently identified, and was further screened to remove proteins with few non-zero peptide hits.

### Preparation of recombinant proteins

The target genes of *B. anthracis* were amplified from its chromosomal DNA by PCR with specific oligonucleotides using a Taq polymerase premix (Invitrogen). Primers used in this study were as follows: GroEL (BA0267), forward GCA AAA GAT ATT AAA TTT AGT GAA, reverse CAT CAT TCC GCC CAT ACC GCC; enolase (BA5364), forward ATG TCA ACA ATT ATT GAT GTT, reverse TCA TCG TTT GAT GTT ATA AAA; and EF-tu (BA0108), forward ATG GCT AAA GCT AAA TTC GAA, reverse TCA CTC AAC GAT AGT AGC AAC. The amplicons were ligated into expression plasmid pTrcHis2-TOPO (Invitrogen) and then transformed into *E. coli* DH5α following the manufacturer's instructions. Protein expression was induced with 1 mM isopropyl-β-D-thiogalactoside for 5 h. The 6× His-tagged fusion proteins were isolated under native conditions by Ni^2+^-NTA resin (Probond, Invitrogen) as described in the manufacturer's protocols. For binding assays, purified proteins (320 µg) were also conjugated to carboxylate-modified FluoSpheres (1.0 µm, 500 µl) in the presence of EDAC (1-ethyl-3-(3-dimethylaminopropyl)-carbodiimide) according to the manufacturer's recommendations (Invitrogen). The resulting beads were blocked with 1% BSA and resuspended in 500 µl of PBS.

### Exosporium extraction and ligand blot analysis

Exosporium extracts were prepared by incubating the spore suspension in 0.1M DTT, 0.05% SDS, and 0.1M NaCl, pH 10 for 2.5 h in a 37°C shaking water bath [27], or by a sonication in 20 mM Tris-HCl, 0.5 mM EDTA, pH 7.5. Sonication was performed using a Microsonix XL ultrasonic cell disruptor (Microson) for seven 1 min bursts (output power 12 W), each separated by 2 min cooling on ice [28]. By centrifugation, spores (exosporium negative) and supernatants were separated. The spore pellets were washed twice with the spore binding buffer as described above, and were subjected to PLG binding assays. For ligand blot analysis, exosporium extracts or recombinant proteins were run on 4–12% SDS-PAGE gel and then electrophoretically transferred onto a nitrocellulose membrane. The membrane was soaked in PBS/0.05 Tween 20 (PBST) containing 1% bovine serum albumin overnight at 4°C to renature the proteins, and then it was incubated with PLG (1 µg/ml in PBST/1% BSA) for 1.5 h at room temperature. The membrane was washed 5 times with PBST and incubated with anti-PLG antibody for 1 h followed by the corresponding horseradish peroxidase (HRP)-conjugated secondary antibody. The blot was visualized by HRP reaction.

### PLG binding to recombinant receptors

MaxiSorp 96 well plates (Nunc) were coated overnight with different concentrations of recombinant proteins (7.8–500 nM) at 4°C. Following 3 washings with PBST, wells were blocked for 1 h at room temperature with 0.1% gelatin/PBS and then washed 3 times. Afterwards, 100 µl/well of human PLG (1 µg/ml) were added and incubated for 2 h at room temperature. Unbound PLG was removed by washing 3 times with PBST. Bound PLG was incubated with anti-PLG antibody (1∶5,000) for 1 h followed by secondary antibody. PLG binding to receptors was colorimetrically measured at 450 nm after sequential addition of a HRP substrate TMB (3,3′,5,5′-tetramethylbenzidine) and sulfuric acid.

### C3 deposition and degradation on spore surface

Spores (2×10^7^/well) were washed, resuspended in PBS, and immobilized onto MaxiSorp microplates (Nunc) overnight at 4°C. After washing with PBST, wells were blocked with PBS/0.2% gelatin for 1 h at room temperature and incubated with 10% NHS (100 µl) for 30 min at room temperature. The wells were washed 2 times with PBST and incubated with 2 µg/well of PLG for 1 h in the presence or absence of protease cocktail (100-fold dilution, Sigma). Bound PLG was activated by uPA (20 units/well) for 3 h at 37°C. Deposited C3b was then detected by incubation with anti-C3c antibody (1∶2,000) followed by HRP-conjugated secondary antibody. C3 deposition was colorimetrically measured after addition of TMB and sulfuric acid at 450 nm.

### Preparation of rabbit BALF and phagocytosis assays

BALF was collected from New Zealand White rabbits infused with 30–40 ml of Hanks' balanced salt solution (HBSS) under the approval of the Institutional Animal Care and Use Committee of the Biocon (Rockville, MD; approval # A0900-09a). The BALF was used after centrifuging at 1,500 rpm for 20 min at 4°C. For macrophage phagocytosis assays, spores were incubated with 250 µg of BALF and/or 25 µg of NHS (as a source of C3) for 1 h at room temperature in the presence or absence of 100 µM leupeptin. Spores (8×10^6^) were washed twice with PBS and resuspended in PBS. RAW264.7 cells were infected with the spores at MOI of 10 and centrifuged to precipitate spores for 2 min. After 30 min of incubation, the cells were washed 6 times with HBSS and lysed by 2.5% saponin, and phagocytosed spores were counted by a serial dilution method on LB agar plate.

### Protein staining and immunoblotting

Proteins were loaded onto 10% or 4–12% NuPAGE MES gel (Invitrogen) and separated under reducing conditions (32 mM dithiothreitol). Separated proteins were then silver-stained using GelCode SilverSNAP kit (Pierce) according to manufacturer's instructions or immunoblotting. For immunoblotting, the separated proteins were electrophoretically transferred to a nitrocellulose membrane using an iBlot gel transfer system (Invitrogen). After blocking with 5% dried milk solution, the membrane was probed with the primary antibody using PBST containing 5% milk, and was incubated with the corresponding HRP-coupled secondary antibody (1∶10,000 dilution) for 1 h at room temperature. Then the membrane was washed in PBST and visualized with the most sensitive West Femto chemiluminescent substrate system (Thermo Scientific).

### Statistical analysis

P-values were calculated by the paired student's t-test. Statistical significance was determined by analysis of variance (ANOVA) prior to Student's t-test. Significance was set at P-values less than 0.05. Error bars in all the figures indicate standard error of the mean (SEM) in a two-tailed t-test.

## Results

### Identification of human serum proteins interacting with *B. anthracis* spores

**Figure 1:**
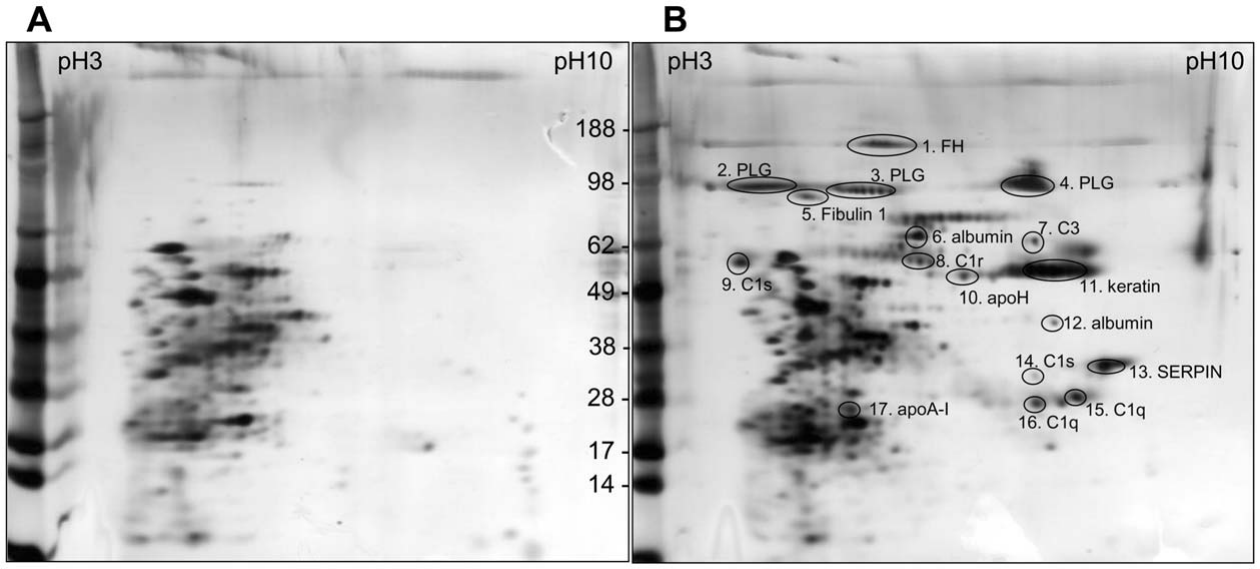
2D gel analysis of human serum proteins interacting with *B. anthracis* Sterne spores. Proteins were eluted from NHS-treated spores and separated by 2D electrophoresis. Proteins were visualized by silver staining and were identified by LTQ-MS/MS as described in Materials and Methods. (A) Control eluates from spores alone without NHS. (B) Proteins eluted from NHS-treated spores by a chaotropic reagent. Protein identifications by LTQ-tandem MS/MS were indicated in gel B.

Spores of the Sterne strain 34F2 were incubated with NHS. After an extensive wash, the bound proteins were eluted by a chaotropic solution followed by desalting. Desalted spore-bound proteins were separated by 2D electrophoresis and visualized by silver staining (Figure 1). Protein spots were excised from the gel and trypsinized to be identified by LC-MS/MS (Table S1). The identified proteins included complement, acute phase proteins, and proteases. Among them, complement factor H and PLG were relatively abundant spore-bound proteins (Figure 1B). To support this finding, we trypsinized total eluted proteins from the spore surface and submitted them to LC-MS/MS. As shown in Table S2, PLG and complements factor H and C3 were abundant among the spore-bound proteins, with high numbers of peptide hits and high peptide identification scores.

### PLG binding to spores is exosporium-dependent

**Figure 2:**
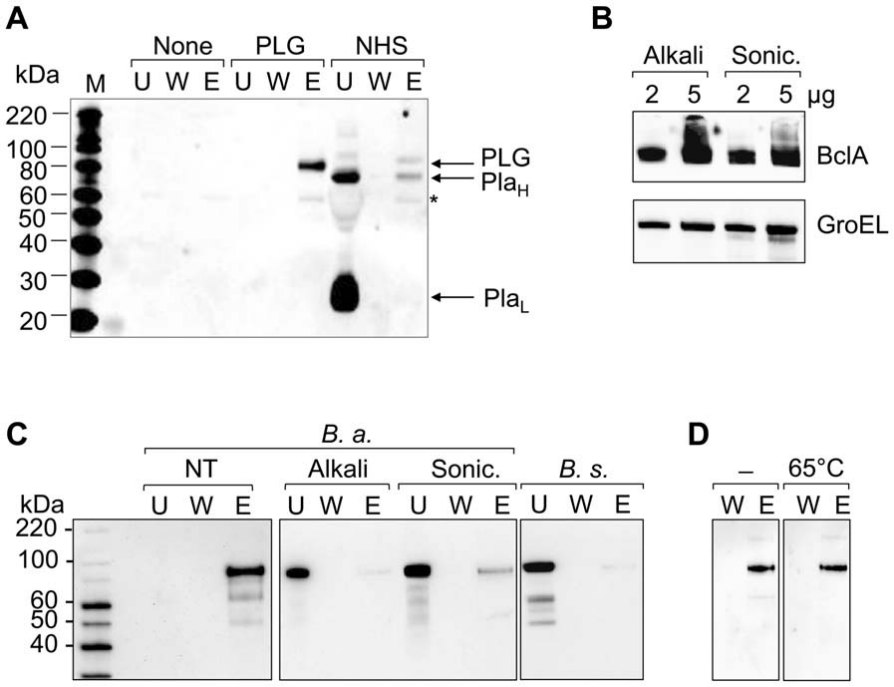
Binding of PLG to *B. anthracis* spores is exosporium-dependent. (A) Spores (1.6×10^9^) were incubated with PLG (10 µg) or NHS (150 µl) and washed, and bound proteins were eluted by 3 M potassium thiocyanate. (A) Western blot of 5^th^ washed (*W*) and eluted (*E*) fractions with anti-PLG antibody. Lane *W* demonstrates an absence of detectable protein in the last wash before elution. *U* indicates unbound proteins. Pla_H_ and Pla_L_ represent heavy chain and light chain of plasmin, respectively. (B) Exosporium was removed by alkaline DTT-SDS-NaCl buffer or sonication as described in Materials and Methods. Removal of exosporium was confirmed by Western blot analysis of major exosporium proteins BclA and GroEL. (C) Exosporium-positive (NT), exosporium-negative (by alkaline extraction or sonication), or *B. subtilis* 168 spores were incubated with PLG and eluted by a chaotropic reagent. Bound PLG was analyzed by Western blotting as described. *U*, unbound; *W*, 5^th^ wash; and *E*, elution with a chaotropic salt. (D) *B. anthracis* spores were incubated at 65°C for 30 min, and subjected to PLG binding assay as described above. No significant change of PLG binding by heat treatment (65°C) was seen compared with no heat treated control (-).

We further examined whether PLG binds to the spore surface of *B. anthracis* using Western blotting. Purified PLG bound efficiently to the spore surface, while plasmin showed more efficient binding in the NHS (Figure 2A), suggesting less existence of PLG in the NHS. To examine whether PLG binding to the spores was exosporium-dependent, we extracted exosporium of spores using an alkaline buffer containing DTT-SDS-NaCl and sonication. To test for the removal of exosporium proteins, we carried out Western blot analysis with antibodies against BclA and GroEL, major exosporium proteins. BclA and GroEL were efficiently extracted by both alkaline solution and sonication (Figure 2B). The resulting exosporium-negative spores were then subjected to the PLG binding assays. Removal of exosporium revealed a significant decrease in PLG binding onto the surfaces (Figure 2C). No detectable amount of PLG was bound in the exosporium-negative, non-pathogenic *B. subtilis* as well. Heat treatment of spores (65°C for 30 min) did not show a significant change PLG binding (Figure 2D), suggesting heat-insensitive receptor-mediated PLG binding. These data demonstrated that exosporium was involved in PLG binding to spores.

### *B. anthracis* spore-bound PLG is functional

**Figure 3:**
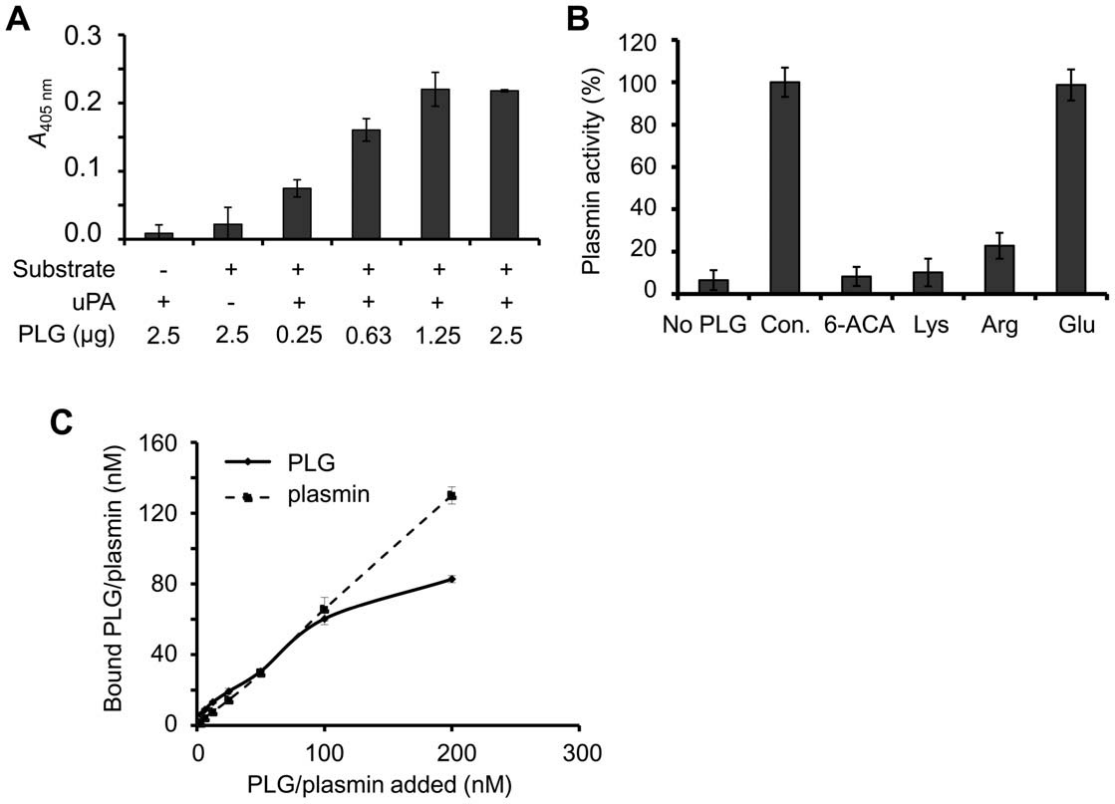
Spore-bound PLG is activated by uPA. (A) Plasmin activity of bound PLG after uPA activation. Spores (5×10^7^) were incubated with PLG and washed, and 20 units of uPA and plasmin substrate VLK-pNA were added. After incubation for 15 min at room temperature, the absorbance was measured at 405 nm. (B) Effect of lysine analogues on spore-bound PLG/plasmin activity. Spores (5×10^7^) were incubated with PLG (2 µg) in the presence of 50 mM amino acids, and assayed as described in *panel A*. (C) Binding affinity of PLG and plasmin to spores. PLG and plasmin were incubated with spores (2.4×10^7^) and the amount of bound proteins was assayed by the colorimetric method described in panel B. The amount of plasmin was determined using a standard activity curve.

To examine whether PLG bound to pathogen receptors can be activated to plasmin, spores were incubated with PLG and its activator uPA. Spore-bound PLG was efficiently activated by uPA in a concentration-dependent manner (Figure 3A). This PLG binding to pathogen surface was abrogated by 50 mM of lysine analogues such as arginine and 6-amino-n-caproic acid (6-ACA), indicating that binding of PLG to spores was specific to the presence of lysine residues in the spore surface proteins (Figure 3B). To compare the binding specificity of PLG and plasmin, purified enzymes were incubated with the spores. The amount of bound proteins after elution and PLG activation by uPA was measured using a colorimetric assay. The assay demonstrated a similar affinity of the spores for PLG and plasmin, within less than 100 nM (Figure 3C).

### Identification of the spore PLG receptors

**Figure 4:**
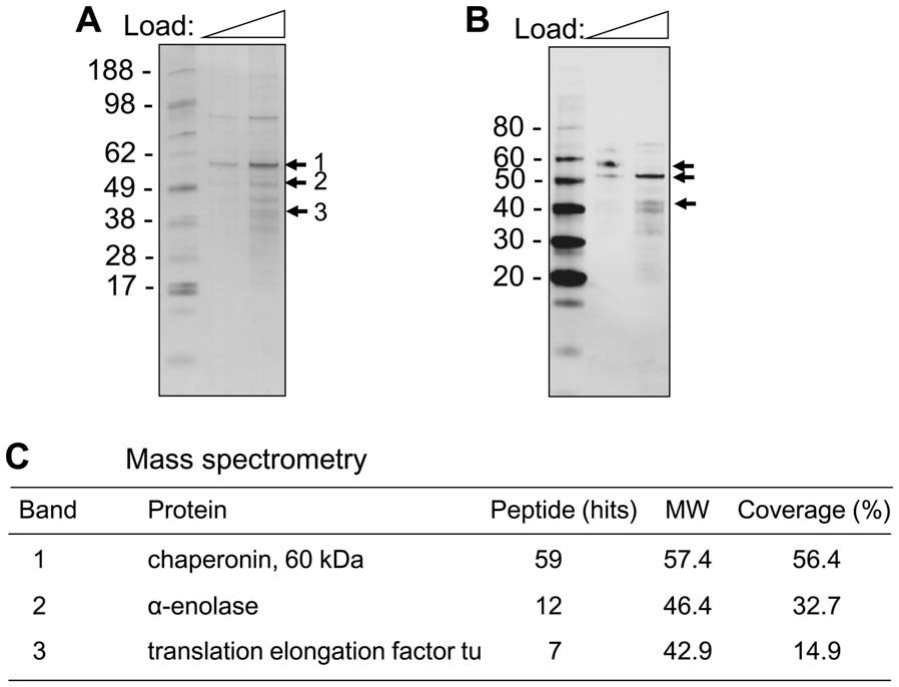
Identification of PLG receptors. Spore extracts obtained from an acidic buffer were separated on a 4–12% SDS-PAGE. (A) The proteins were visualized by coomassie brilliant blue staining (CBB). (B) Far-Western (Ligand) blot with PLG. The proteins were transferred onto a nitrocellulose membrane, and the membrane was incubated with PLG. PLG-bound proteins were visualized by Western blotting with anti-PLG antibody. (C) Identification of proteins interacting with PLG. Bands (1–3) were excised from the gel and subjected to in-gel digestion with trypsin. LTQ-MS/MS was performed to identify the proteins.

**Figure 5:**
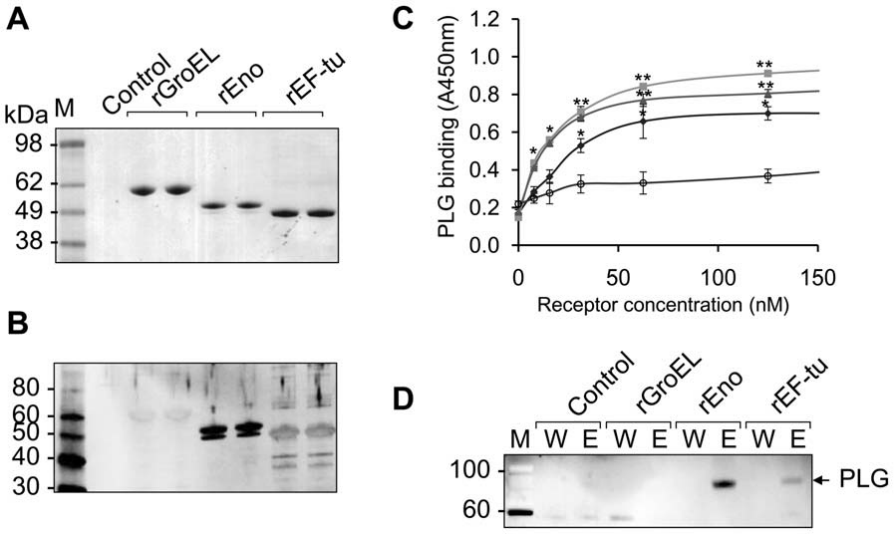
PLG binding of recombinant receptors. (A) Recombinant proteins were expressed on plasmid pTrcHis2-TOPO by IPTG induction and purified by Ni^+^-chelate column chromatography. The purified proteins were visualized on SDS-PAGE by Coomassie blue staining. (B) Analysis of PLG binding of recombinant receptors by ligand blot. The bound PLG was detected by anti-PLG antibody. (C) Analysis of PLG binding of recombinant receptors by ELISA. Different concentrations of receptors were immobilized on a plate. After incubation with PLG (0.1 µg/well), binding activity was analyzed by anti-PLG antibody antibody. ▪, enolase; ▴, EF-tu; •, GroEL; and ○, control BSA. (D) PLG binding of receptor-conjugated beads. Receptor-conjugated beads (50 µl) were incubated with PLG (1 µg) and eluted with 100 µl of 3 M potassium thiocyanate. The eluted protein (10 µl) from the beads was analyzed by Western blotting with anti-PLG.

To screen for the PLG receptors, exosporium extracts were prepared in an alkaline buffer as described above and subjected to a ligand blot analysis overlaid with PLG and probed with anti-PLG antibody. The bands of silver-stained gel (Figure 4A) corresponding to each PLG binding protein in a ligand blot (Figure 4B) were excised from the gel and subjected to tryptic digestion and peptide mass fingerprinting using a LTQ-tandem MS/MS. Bands 1–3 were identified to be chaperonin-60 kDa (GroEL), α-enolase, and translation elongation factor-tu (EF-tu), respectively (Figure 4C). To further confirm the putative PLG receptors in experiments with recombinant proteins, the genes of the above proteins were PCR-amplified from a bacterial chromosome using specific primers. The amplicons were inserted into pTrcHis-TOPO vector to generate proteins and all recombinant proteins were expressed in *E. coli* and purified (Figure 5A). Strong PLG binding to α-enolase and EF-tu, but weak binding to GroEL, was observed in a ligand blot assay (Figure 5B). Binding affinity of recombinant receptors to PLG was further determined in a microplate coated with different concentrations of receptor proteins. PLG binding to the receptors was dose-dependent and the affinity was in the order of α-enolase>EF-tu>GroEL (Figure 5C). To further support the differential binding, we conjugated beads (particle diameter of 1 µm) with recombinant proteins to mimic spores. PLG was able to bind strongly with α-enolase-conjugated beads and weakly with EF-tu-congugated beads (Figure 5D). Together, these results suggest that α-enolase and EF-tu are PLG receptors of *B. anthracis* spores.

### Surface-bound PLG exhibits anti-opsonic activities

**Figure 6:**
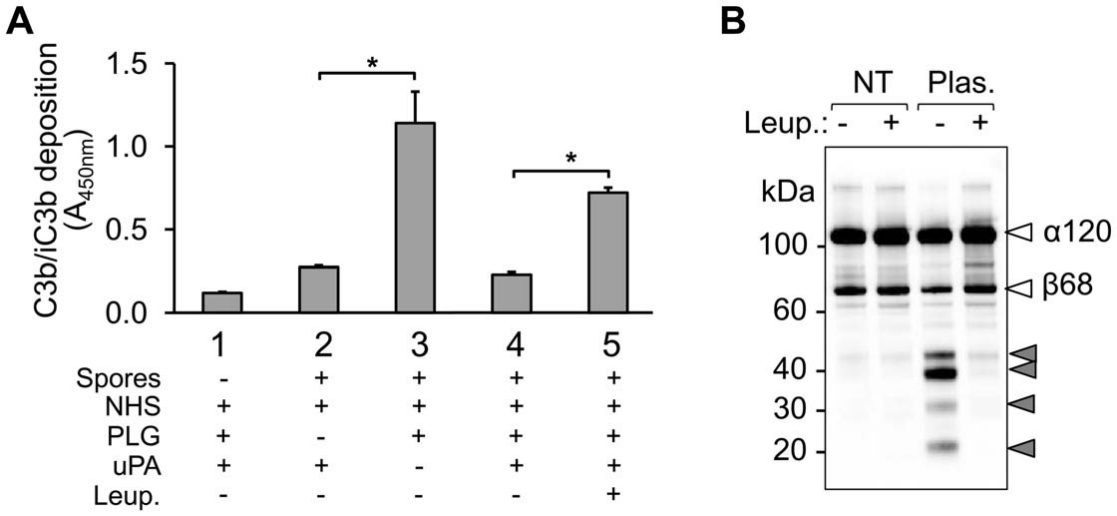
Degradation of deposited C3b by *B. anthracis* spores-bound PLG. (A) Spores (2×10^7^/well) were immobilized onto a microplate and incubated with 10% NHS (100 µl) for 30 min at room temperature. Washed spores were incubated with 2 µg/well PLG for 1 h in the presence or absence of protease cocktail. Bound PLG was activated by uPA (20 units/well) for 3 h at 37°C. Deposited C3b was detected by anti-C3c IgG and HRP-conjugated secondary antibody followed by TMB reaction. C3b deposition is expressed as the mean absorbance at 450 nm of quadruplicates. Error bars indicate ± standard deviation. *P<0.001 (paired Student's t-test). (B) Spores were incubated with plasmin and washed extensively as described in Figure 2A. C3b was incubated with the spores in the presence or the absence of leupeptin and C3b degradation was analyzed by Western blot with anti-C3c antibody.

It has been known that upon complement activation, C3b, along with cleavage products such as iC3b, is covalently attached to target surfaces to opsonize the pathogenic organisms for pathogenesis [29], [30]. To assess whether spore-bound PLG is able to degrade C3b deposited on the surface, we performed a C3b deposition and degradation assay based on whole-spore ELISA. Spores were immobilized onto microplates and treated with NHS and PLG. After extensive washing, spore-bound PLG was activated by uPA, and deposition of C3b on the spore surface was monitored. Spore-bound active plasmin led to a drastic decrease in C3b molecules on the surface compared to control without PLG activation by uPA, or those pretreated with PLG in the presence of protease cocktail (Figure 6A). Plasmin-mediated C3b degradation was confirmed by Western blot analysis after incubation of C3b with plasmin-coated spores. Both α and β chain of C3b were degraded by spore-coated plasmin, which was inhibited by leupeptin treatment (Figure 6B). These suggest that after its activation by uPA, spore surface-bound PLG exhibits anti-opsonic activity by cleaving C3b molecules.

### Rabbit BALF decreases complement-dependent phagocytosis

**Figure 7:**
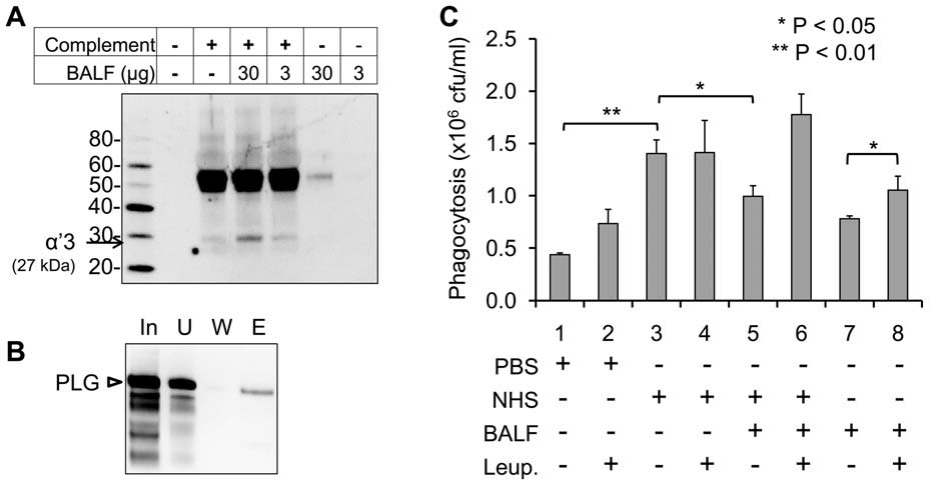
Rabbit BALF decreases spore phagocytosis of macrophages. (A) Rabbit complements were incubated with BALF (3 and 30 µg) in the PBS for 1 h and subjected to Western blot analysis with anti-C3b antibody. Degradation of C3b was indicated by arrow (27 kDa of α′3 chain). (B) PLG in the BALF interacts with spores. BALF was incubated with spores, eluted by a chaotropic salt and subjected to Western blot analysis with anti-rabbit PLG antibody. (C) BALF decreases NHS-mediated spore phagocytosis. RAW264.7 cells were infected with NHS-treated spores in the presence of BALF and/or leupeptin. Phagocytosed spores were determined by a serial agar plating method.

In inhalation anthrax, the lung serves as a portal of entry. Before alveolar macrophages phagocytose deposited spores, they may interact with the BALF in the alveolar space. Therefore, we examined whether the interaction with BALF increases or decreases spore phagocytosis. When rabbit complements were incubated with the BALF, degraded C3b fragment was detected in Western blot with anti-C3 antibody, suggesting the existence of C3 degrading protein(s) (Figure 7A). To confirm the existence of PLG and its binding to spores, spores were incubated with BALF and the bound proteins were submitted to Western blot analysis with anti-PLG antibody. As shown in Figure 7B, PLG in the rabbit BALF bound to spores. To evaluate the role of spore-bound PLG on the phagocytosis, we incubated *B. anthracis* spores with BALF without activation of PLG by uPA in order to examine the intact BALF effects. Murine macrophages RAW264.7 cells were then infected with the spores in the presence of NHS. The macrophages were lysed and the number of viable bacteria was determined by counting CFU following dilution plating. Phagocytosis in the presence of NHS alone increased the CFU 3-fold per well at 30 min post-infection and decreased the CFU per well in the presence of NHS and BALF (Figure 7C). A decrease was observed in the NHS only treated samples when leupeptin, a plasmin inhibitor, was amended to the exposure media. Taken together, PLG in the BALF may play a role in host defense in the lung.

## Discussion

Following a proteomic approach, we have found that *B. anthracis* spores bind a number of human serum proteins that are involved in humoral and innate immunity. Spore-bound proteins included complement proteins, blood coagulation/fibrinolysis regulators, acute phase proteins, and cell surface and extracellular proteins. Deposition of complement C3 onto *B. anthracis* spores has been reported to be required for opsonin-dependent phagocytosis by macrophages [21]. Inhaled pathogens such as *Mycobacterium tuberculosis* [31], [32] and *Pseudomonas aeruginosa* [33] were phagocytosed by macrophages in a C3-dependent manner to be subsequently cleared by the lungs. Although C3 opsonization of pathogens including *B. anthracis* facilitates early infection steps [16], [20], [21], the pathogens inhibit the host complement attack and apparently utilize diverse escape mechanisms. Our proteomic data suggest that complement regulators are involved in control of complement activation. Complement factor H, FHR-1, C1 inhibitor, and C4BP were acquired by *B. anthracis* spores (Table S2). Complement factor H, a 150-kDa plasma glycoprotein, is the central fluid-phase regulator of the alternative complement pathway. Further study on complement factor H -mediated C3b opsonization is warranted. In the present study, we focused on whether spore-bound PLG regulates spore opsonization by C3b. The results showed that spore-bound PLG induces a significant decrease in C3b opsonization by uPA activation. Therefore, it is likely that the acquisition of host regulators masks the pathogenic surface, resulting in survival of *B. anthracis* spores. This might represent a novel mechanism to inhibit the host innate immune system during early *B. anthracis* infection.

Acquisition of PLG and its subsequent conversion to active plasmin promote dissemination of bacteria in the host [34]. Several invasive bacterial pathogens utilize the PLG system to invade tissues [16], [35], [36], and use of this system has been extended to viruses [37] and parasites [38]. Increasing evidence proposes a so-called “bacterial metastasis” that is facilitated by the binding and activation of PLG and by the colonization and invasion of PLG-bound bacteria into tissues [35].

As shown in this study, *B. anthracis* is capable of binding PLG on the outer surface of spores. The bound PLG is activated to plasmin by the addition of human activator uPA, promoting a surface-associated plasmin activity. The binding and activation are inhibited by the presence of the lysine analogue 6-ACA, suggesting a lysine-dependent binding of PLG to spores. This supports the existence of lysine binding sites in PLG, which has been shown for several pathogens to be mediated by PLG kringle domains [39]–[41]. This may account for the similar binding capacity to spore receptors in spite of the conformational change by proteolytic cleavage between the kringle and protease domains [42].

Heat-resistant spores of *B. anthracis* were retained in the lungs of mice challenged with aerosolized Sterne spores for all infection periods [43]. Unlike the lung, homogenates of other organs such as lymph node, liver, and spleen showed the presence only of vegetative bacilli in the same experiment [43]. Accordingly, spores are the first cells of *B. anthracis* which invade through lung barriers, and vegetative cells are the type that circulate in the bloodstream and invade organs. The binding of host PLG or plasmin might represent a mechanism to regulate several physiological processes, e.g., fibrinolysis, ECM degradation, cell migration, the processing of growth factors, and bacterial metastasis into several organs [44].

The Gram-positive bacteria group A streptococci interact with PLG via GAPDH and α–enolase on the surface [45], [46]. Proteomic analysis of the PLG-binding proteins in the human pathogen *M. tuberculosis* identified glutamine synthase A1, HSP70 (DnaK), HSP60 (groEL), EF-tu, and other proteins that are metabolic proteins and are localized extracellularly [47]–[49]. In our study, PLG-binding proteins of *B. anthracis* spores were identified by ligand Western blotting of spore exosporium extracts to be GroEL, α-enolase, and EF-tu. They are extracellularly localized, consistent with those of other human pathogens. Since a non-virulent *B. subtilis* has no exosporium, these PLG receptors might not be localized in outmost surface of its spores resulting in no PLG binding as shown in Figure 2. This could be a feature characteristic of a virulent *B. anthracis* in contrast to a non-virulent *B. subtilis*. The PLG binding to receptor proteins is usually mediated by a carboxyl-terminal lysine residue. In a competition assay, lysine and its analogue 6-ACA significantly inhibited PLG binding to spores, suggesting that a lysine residue is involved in PLG binding.

Although spore- or cell-bound PLG is activated by the host PLG activation system, some pathogens present an endogenous PLG activation system, e.g., streptokinase [50], staphylokinase [51], and Pla [52]. *B. anthracis* secretes two major metalloproteases, Npr599 (or NprB) and InhA, that exhibit PLG-degrading activity [15]. However, proteolysis of PLG by these proteases did not display plasmin activity [15]. This activity has been shown in the NprB homologue bacillolysin MA, produced by *B. megaterium*, which converts PLG into angiostatin and mini-PLG [53]. Another NprB-like protease, aureolysin of *S. aureus*, has the ability to convert PLG into angiostatin and mini-PLG and activates pro-uPA to uPA [54]. This observation opens up the possibility that in addition to the host activation, *B. anthracis*-bound PLG may be activated by a bacterial activation system such as pro-uPA activation by bacterial proteases. Thus, whether PLG activation during *B. anthracis* infection is due to secreted proteases, the host PLG activation system, or both deserves further study.

Based on the above considerations, complement regulators from bacterial pathogens could be vaccine candidates to fight bacterial infections. For instance, GNA1870, a lipoprotein of *Neisseria meningitidis*, was identified to be a complement factor H binding protein and meningococcal vaccine candidate [55]. In animal models, antibodies binding to GNA1870 inhibit binding of complement factor H and thus render the bacterium susceptible to the alternative complement pathway. Furthermore, the bound antibodies activate the classical pathway, thereby initiating and enhancing complement attack [55]. PLG binding proteins as regulators of complement C3b might be useful as additional vaccine targets to avoid the immune evasion by the above discussed mechanisms. A recent study showed that *Streptococcus suis* enolase localizes on the cell surface and facilitates bacterial adherence, and that enolase confers complete protection against infection to mice [56]. Proteomic analysis showed that *B. anthracis* expresses α-enolase as a dominant immunogenic antigen [57], [58]; however, the function of enolase has not been established. Another study demonstrated that *B. anthracis* glyceraldehydes-3-phosphate was predominantly interacted with plasminogen and that immunization with the recombinant protein resulted in a significant protection upon challenge with *B. anthracis* in the murine model [25]. Therefore, we propose that *B. anthracis* proteins interacting with PLG (i.e. α-enolase) function as a protective antigen and are vaccine candidates to inhibit innate immune evasion by the pathogen.

In summary, we have shown that *B. anthracis* may utilize the host PLG system to regulate complement opsonization in order to evade innate immunity as reported elsewhere [17], [31], [35], [59], [60]. We have identified PLG as a spore-associating protein. PLG-bound spores were capable of exhibiting anti-opsonic properties by cleaving C3b molecules through the surface receptors α-enolase and EF-tu. PLG-dependent anti-oposonization was confirmed in the rabbit BALF by its C3b degradation and anti-phagocytic activity. This suggests new avenues for development of anti-opsonization agents with capacity in affecting innate immunity.

## Supporting Information

Table S1 **Identification of human serum proteins interacting with** ***B. anthracis*** **spores separated by 2D SDS-PAGE.** Spore-bound serum proteins were separated onto 2D SDS-PAGE and the each spot excised from the gel was subjected to LTQ-MS/MS.
(XLS)

Table S2 **Identification of human serum proteins interacting with** ***B. anthracis*** **spores.** Spore-bound serum proteins were subjected to LTQ-MS/MS. Percentage of wrong identification was estimated to be ∼2%, and #IDs ≥5 are listed. #Peptides and #IDs represent the number of different peptides detected from this protein and the total number of peptide identifications from this protein, respectively.
(XLS)
